# Self-Healing and Tough Polyacrylic Acid-Based Hydrogels for Micro-Strain Sensors

**DOI:** 10.3390/gels11070475

**Published:** 2025-06-20

**Authors:** Chuanjie Liu, Zhihong Liu, Bing Lu

**Affiliations:** 1 School of Chemistry and Chemical Engineering, Beijing Institute of Technology, Beijing 100081, China; 7520230176@bit.edu.cn; 2 School of Digital Media, Shenzhen Polytechnic University, Shenzhen 518055, China

**Keywords:** high strength and toughness, self-healing, micro-strain, sensing

## Abstract

Self-healing hydrogels hold promise for smart sensors in bioengineering and intelligent systems, yet balancing self-healing ability with mechanical strength remains challenging. In this study, a self-healing hydrogel exhibiting superior stretchability was developed by embedding a combination of hydrogen bonding and dynamic metal coordination interactions, introduced by modified fenugreek galactomannan, ferric ions, and lignin silver nanoparticles, into a covalent polyacrylic acid (PAA) matrix. Synergistic covalent and multiple non-covalent interactions enabled the hydrogel with high self-healing ability and enhanced mechanical property. In particular, due to the introduction of multiple energy dissipation mechanisms, particularly migrative dynamic metal coordination interactions, the hydrogel exhibited ultra-high stretchability of up to 2000%. Furthermore, with the incorporation of lignin silver nanoparticles and ferric ions, the hydrogel demonstrated excellent strain sensitivity (gauge factor ≈ 3.94), with stable and repeatable resistance signals. Assembled into a flexible strain sensor, it effectively detected subtle human motions and organ vibrations, and even replaced conductive rubber in gaming controllers for real-time inputs. This study provides a versatile strategy for designing multifunctional hydrogels for advanced sensing applications.

## 1. Introduction

Flexible and wearable micro-sensors have attracted significant attention in recent years due to their vast potential in applications such as health monitoring, human–machine interfaces, and soft robotics [1,2,3,4]. To realize their full potential, the development of advanced sensing materials must seamlessly integrate mechanical flexibility with high sensitivity, ensuring reliable performance under dynamic and complex deformation conditions [5,6]. Hydrogels are three-dimensional (3D) network structures of chemically or physically crosslinked polymers with high water content, excellent flexibility and stretchability, making them potential candidates for bioengineering, expandable skin sensors, medical health monitoring, and soft robots [7,8,9,10]. However, conventional hydrogels often suffer from limitations such as poor mechanical robustness, low stretchability, and irreversible damage upon mechanical failure, which severely restrict their practical use in dynamic and demanding environments.

To address these challenges, recent research has focused on the development of tough and highly stretchable hydrogels with self-healing capabilities. Such hydrogels can maintain mechanical integrity under large deformations and autonomously restore their structure and functionality after damage without external stimuli, significantly enhancing device durability and lifespan [11,12]. This ability enables long-term, sustainable sensor operation, extends its service lifespan, and maintaining its strain sensitivity [13,14,15].

In recent years, non-covalent interactions, including metal–ligand, host–guest [16], hydrogen bonding [17], and hydrophobic interactions [18], have been introduced into the synthesis of self-healing hydrogels. In this context, metal–ligand interaction is an important dynamic non-covalent interaction and can be used as a physical cross-linker to prepare high-strength hydrogels. Through the dynamic ionic bonding and migration of metal ions, metal–ligand interactions can automatically heal, thereby preparing self-healing hydrogels [19,20]. Nevertheless, most previously reported self-healing sensors usually have difficulty in combining ideal mechanical properties, self-healing ability, and micro-strain sensing ability, which greatly limits the practical application of hydrogel sensors. Modified fenugreek galactomannan (OFG) is a natural polysaccharide derived from fenugreek seeds, chemically modified to enhance its solubility, water absorption, and mechanical properties [21,22]. When incorporated into hydrogels, OFG forms hydrogen bonds and dynamic non-covalent interactions with other components, such as PAA and metal ions, enabling the hydrogel to self-heal and improving its mechanical strength and flexibility.

In addition to mechanical resilience, the electrical sensitivity of hydrogels—particularly their resistance changes in response to mechanical stimuli—is critical for sensor performance. High gauge sensitivity allows for accurate and real-time monitoring of strain, pressure, and motion, which is essential for micro-scale sensing systems [23,24]. Therefore, developing multifunctional hydrogels that simultaneously integrate high toughness, large stretchability, self-healing ability, and pronounced piezoresistive responsiveness is of great significance.

In this study, a self-healing PAA/OFG-Fe^3+^ hydrogel (SPH) with excellent mechanical properties was developed by introducing multiple non-covalent interactions into a covalently crosslinked PAA network. Uniform and extensive H-bonding occurred between OFG, the PAA network, and lignin silver nanoparticles (Ag-NPs). In particular, dynamic metal coordination interactions played a key role in the development of the hydrogel, with Fe^3+^ forming coordination bonds with the carboxyl groups on the PAA/OFG matrix. The slippable dynamic metal coordination interactions allowed the hydrogel to achieve autonomous self-healing and exceptional mechanical strength (stretchability up to 2000%, fracture strength > 130 kPa). Furthermore, with the incorporation of conductive Ag-NPs and Fe^3+^, the hydrogel demonstrated excellent strain sensitivity (gauge factor ≈ 3.94), along with repeatable and accurate resistance signal responses. Leveraging these advantages, the hydrogel was successfully assembled into a flexible strain sensor capable of monitoring subtle human motions and vocal vibrations of organs. This work offers a promising strategy for enriching the design and application of multifunctional hydrogels.

## 2. Results and Discussion

### 2.1. Characterization Results of the Hydrogel

**Figure 1:**
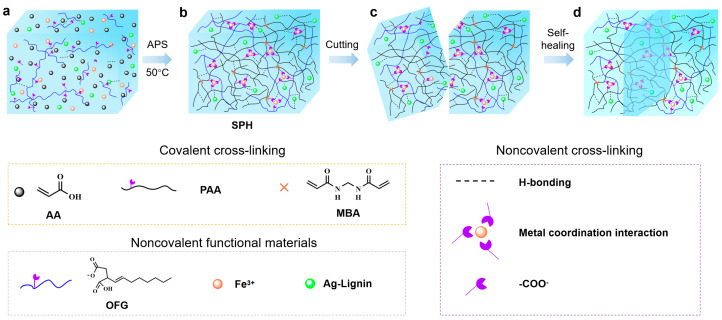
Synthesis and self-healing mechanism of the hydrogel. (**a**,**b**) Synthesis of the hydrogel. (**c**,**d**) Schematic diagram of the self-healing process of the hydrogel.

The preparation mechanism of the hydrogel proposed in this paper is shown in Figure 1. A hydrogel with uniform texture and good performance was prepared by a one-pot polymerization method. The acrylic acid (AA) monomers undergo free radical polymerization under the action of the initiator ammonium persulfate (APS) and cross-linker N,N’-methylenebisacrylamide (MBA) to form covalent PAA network. The abundant carboxyl groups (–COOH) existing in PAA and OFG chains and hydroxyl (–OH) contributed to numerous H-bonds.

**Figure 2:**
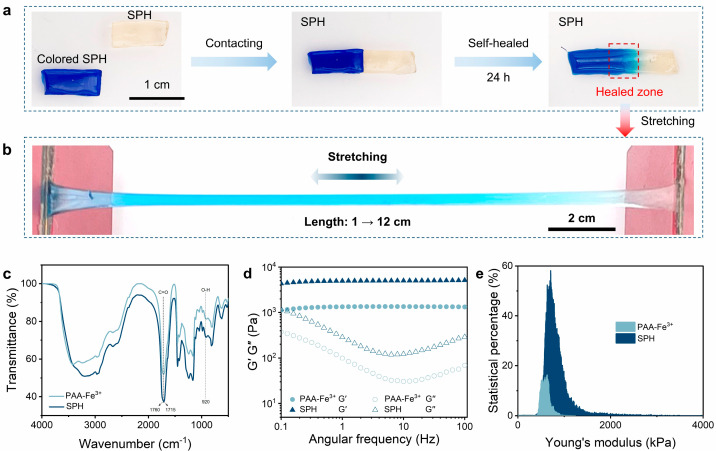
Characterization of PAA-Fe^3+^ and SPH. (**a**) Self-healing process of the hydrogel. Two hydrogels of different colors were placed at room temperature for 24 h. (**b**) The healed hydrogel being stretched. (**c**) FTIR. (**d**) Storage modulus (G’) and loss modulus (G’’). (**e**) Distribution of Young’s modulus.

Meanwhile, the –COOH functionalities in the PAA and OFG chains engaged in dynamic and reversible coordination interactions with Fe^3+^ ions. Owing to the higher bonding energy compared to hydrogen bonds, metal coordination interaction can enhance the mechanical strength and performance stability of self-healing hydrogels. The self-healing ability and tensile properties of the hydrogel were studied by using hydrogels of different colors and observing their tensile images after recovery. As shown in Figure 2a, the dyed and undyed hydrogel samples were cut into two halves and then brought into contact with each other. After 24 h, the two pieces of hydrogel healed automatically, and the cut disappeared. During the self-healing process, the dye diffused from one piece of hydrogel to the other, resulting in a blurred contact interface between the two pieces of hydrogel, and the healed cut did not break after stretching Figure 2b.

As shown in Figure 2c, the absorption peak of the stretching vibration of the free carboxylic acid O-H is located at 3550 cm^−1^. Due to the formation of the dimer, the carboxyl peak shifts to a lower wavenumber, forming a broad and dispersed peak in the range of 3200–2500 cm^−1^. The formation of the dimer also affects the C=O stretching vibration of the free carboxylic acid, shifting it from 1760 cm^−1^ to 1715 cm^−1^. A peak at 920 cm^−1^ is observed, which is a characteristic peak of the out-of-plane rocking vibration of the two-molecule association of the O-H group. The absorption band at 3424 cm^−1^ is attributed to the symmetric stretching vibration of the O-H stretching peak of the hydrogel, while the O-H stretching peak of SPH shifts to a lower wavenumber of 3400 cm^−1^. According to previous reports [19], the obvious shift in the absorption band to a lower wavenumber indicates the formation of hydrogen bonds between PAA and OFG. Compared with PAA-Fe^3+^, there is no obvious position change in SPH, indicating that the introduction of OFG does not significantly change the structure of the hydrogel. Figure S1 shows the X-ray diffraction pattern of the hydrogel. The X-ray diffraction spectrum of the hydrogel does not show an obvious crystalline phase, and there is a broad peak at about 20°, which is caused by the overall crystallinity of the hydrogel skeleton.

By comparing the rheological data of PAA-Fe^3+^ and SPH, the storage modulus G′ of both hydrogels is higher than the loss modulus G″ during the rheological test (Figure 2d), and the loss factor is appropriate, indicating that their molecular chains have a certain degree of rigidity and flexibility, and an effective cross-linked network is formed between the molecular chains. The cross-linked structure may be relatively regular, enabling the material to exhibit good elasticity under dynamic stress. The dense entanglement of PAA and OFG chains around the cross-linking points of the SPH provides the mechanical strength for the hydrogel. The G′ of SPH is significantly higher than that of PAA-Fe^3+^, and its stress–strain data is discussed and analyzed in the following paragraphs. The change in Young’s modulus of the hydrogel surface before and after the addition of OFG was measured by AFM (Figure 2e). The Young’s modulus of the hydrogel after the addition of OFG increased significantly, which is consistent with the rheological data. Figure S2 shows the scanning electron microscope (SEM) images of the freeze-dried PAA-Fe^3+^ and SPHs; both hydrogels are composed of a cross-linked network and a uniform microporous structure, with a pore size range of 10–100 μm. However, the pore structure of the SPH network is more complex and the pore size is smaller, indicating that the structure of the hydrogel is denser. This is attributed to the formation process of the hydrogel; the strong hydrogen bonds between the functional groups and the metal–ligand interaction between Fe^3+^ and OFG increase the pore density of the hydrogel.

### 2.2. Mechanical Properties of the Hydrogel

**Figure 3:**
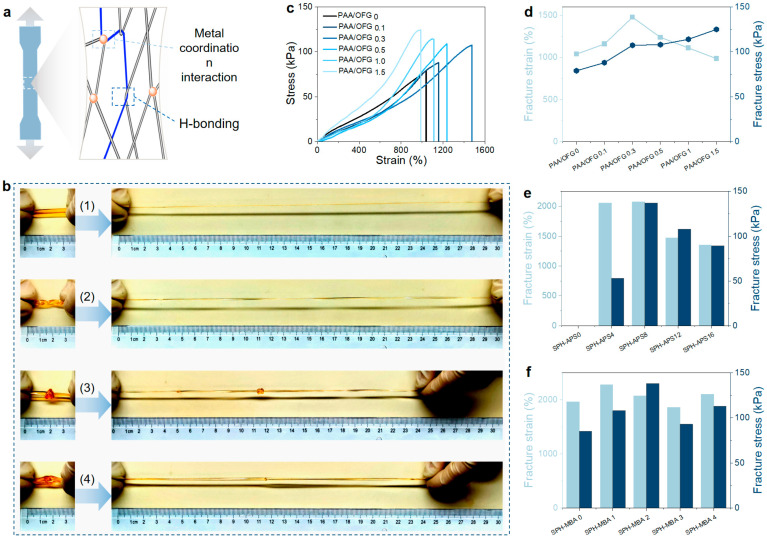
Tensile properties of the hydrogel. (**a**) A stretchable topological network structure composed of cross-linked molecular chains and entangled molecular chains. (**b**) The hydrogel can withstand different forms of stretching (knotting, crossing, and twisting). (**c**,**d**) Influence of the addition of OFG on the mechanical properties of the hydrogel. (**e**) Influence of the addition of APS on the mechanical properties of the hydrogel. (**f**) Influence of the addition of MBA on the mechanical properties of the hydrogel.

We described a rationally designed topological molecular network. PAA and OFG chains are interconnected and entangled through abundant intermolecular hydrogen bonds, electrostatic interactions, and metal coordination interactions, serving as sacrificial conformations for energy dissipation during the deformation of the hydrogel (Figure 3a). As illustrated in Figure 3b, the fabricated hydrogel exhibits outstanding tensile properties, demonstrating the ability to undergo significant deformation, including uniaxial stretching, twisting under tension, and knotting while stretched, with an elongation exceeding ten times its original length. By comparing the rheological data (Figure 2c) and the tensile data (Figure 3), the hydrogel has a relatively high storage modulus and an appropriate loss factor during the rheological test, and the tensile Young’s modulus conforms to Hooke’s law, indicating that its molecular chains have a certain degree of rigidity and flexibility. In order to further explore the influence of the addition amount of each component in the preparation of the hydrogel on its mechanical properties, this paper discussed the influence of the addition amounts of OFG, Fe^3+^, APS, and MBA on the stress–strain of the hydrogel.

Figure 3c–f show the influence of different components on the mechanical properties of the hydrogel to evaluate the optimized content of each component. Firstly, the influence of the OFG content on the mechanical properties of the hydrogel was explored (Figure 3c). As the OFG content increased from 0 to 1.5%, the hydrogel exhibited a peak strain at 0.3% OFG, with a maximum breaking strength and elongation of 120 ± 10 kPa and 1000 ± 53% (*n* = 5), respectively. The highest stretchability and corresponding fracture strength reached 1450 ± 85% and 100 ± 13 kPa (*n* = 5). The mechanical strength of the SPH improved with increasing OFG content, likely due to enhanced hydrogen bonding and metal–ligand coordination. However, beyond 0.3% OFG, the elongation decreased, attributed to excessive non-covalent interactions restricting polymer chain mobility, thereby reducing flexibility and ductility.

Fe^3+^ ions coordinate with –COOH groups on PAA and OFG chains, forming a cross-linked network. Moderate Fe^3+^ addition increases cross-linking density, enhancing the hydrogel’s strength and toughness by promoting uniform stress distribution. However, excessive Fe^3+^ leads to overly dense networks and possible aggregation, reducing chain mobility, increasing brittleness, and creating stress concentration points. As shown in Figure S3, PAA/OFG-Fe^3+^0.10 shows the best mechanical performance.

Within a suitable range, increasing APS content promotes initiator decomposition, generating more free radicals that accelerate acrylic acid polymerization and enhance the cross-linking degree of the hydrogel. A higher cross-linking density contributes to a denser network, improving the hydrogel’s strength, elasticity, and toughness (Figure 3e and Figure S3c). However, excessive APS leads to an overly rapid reaction and high radical concentration, causing issues such as local overheating, uncontrolled polymerization, and structural defects. Moreover, an excessively cross-linked network may become too rigid, compromising flexibility and overall mechanical performance. Residual initiator may also negatively affect the hydrogel’s properties.

Within a suitable range, increasing MBA content enhances the cross-linking density of the polyacrylic acid hydrogel, forming a denser three-dimensional network that improves stress transfer and restricts molecular chain slippage, thereby enhancing strength, hardness, and elastic modulus. Moderate MBA addition significantly improves tensile strength and deformation resistance (Figure 3f and Figure S3d). However, excessive MBA leads to an overly rigid network, limiting chain mobility, reducing flexibility and toughness, and increasing the risk of brittle fracture. Additionally, a high MBA concentration may cause rapid gelation and uneven polymerization, resulting in local stress concentrations and compromised mechanical properties.

Overall, this study obtained an optimized formulation for the preparation of the hydrogel: (m (AA) 6.5 g, m (OFG) 0.09 g, m (FeCl_3_) 0.10 g, m (APS) 8 mg, m (MBA) 2 mg), with stress and strain of 137 ± 17 kPa and 2076 ± 50% (*n* = 5), respectively. The excellent mechanical properties of SPH are derived from its COO^−^/Fe^3+^ coordination network, H-bonding, PAA covalent network, high network density, and chain entanglement.

### 2.3. Self-Healing Properties of the Hydrogel

The hydrogel exhibits excellent self-healing capability, primarily attributed to its flexible polymer network and dynamic non-covalent interactions. On the one hand, the polymer chains possess intrinsic flexibility, allowing for rearrangement and entanglement after damage, which serves as a form of physical cross-linking to partially restore the network and mitigate further failure. On the other hand, dynamic and reversible interactions, such as hydrogen bonding between carboxyl groups and Fe^3+^ coordination, play a key role in healing. Upon fracture, these interactions dissociate at the damaged interface, generating active sites that can re-form under appropriate conditions through chain migration and reorientation. The synergistic effect of hydrogen bonding and metal–ligand coordination significantly enhances healing efficiency and network reconstruction, enabling the hydrogel to rapidly recover its structure and mechanical performance after damage.

**Figure 4:**
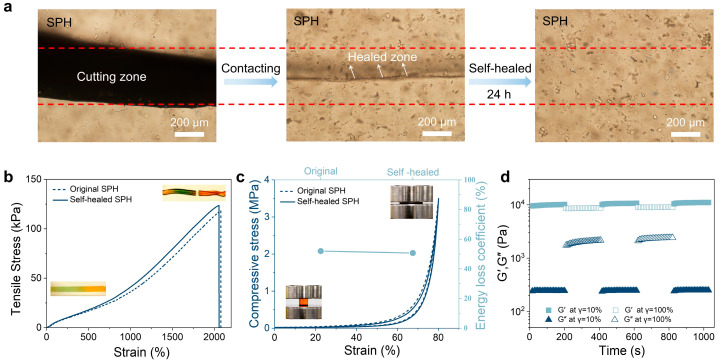
Self-healing properties of the hydrogel. (**a**) Optical microscope images show the changes in the cuts of two pieces of hydrogel over time at room temperature. (**b**) Tensile stress–strain curves of the original hydrogel and the self-healed hydrogel. (**c**) Compressive stress–strain curves and energy loss efficiency of the original hydrogel and the self-healed hydrogel. (**d**) The self-healing properties of the hydrogel are verified by measuring continuous step strains (10% strain→100% strain→10% strain→100% strain→10% strain).

In this study, the self-healing process of the hydrogel cut was observed using an inverted microscope. As shown in Figure 4a, the hydrogel sample was cut into two halves and then brought into contact with each other. After 24 h, the two pieces of hydrogel healed automatically, and the contact interface of the hydrogel became blurred. When stretching the hydrogel (Figure 4b), no cracks were observed, and the rupture site did not coincide with the wound healing site, indicating that the gel had been completely repaired. Generally, the area of the hysteresis loop represents the energy dissipation per unit volume during the deformation process. To evaluate the self-healing performance of SPH, loading and unloading tests were carried out on the prepared hydrogel. As shown in Figure 4c, the hysteresis loops of the hydrogel before and after self-healing were similar, further demonstrating that the hydrogel has remarkable self-healing properties.

The rheological experiment demonstrated that the hydrogel has stable mechanical strength and self-healing properties. Within a fixed time interval of 200 s, the hydrogel was subjected to alternating step strain tests (frequency = 1.0 Hz, strain = 10% or 100%), as shown in Figure 4d. The SPH oscillated under a small amplitude (frequency = 1.0 Hz, strain = 10%), with G′ being approximately 10.0 kPa, G″ being approximately 0.24 kPa (Tanδ = G″/G′ ≈ 0.02); when a large-amplitude oscillating force was applied to the hydrogel (frequency = 1.0 Hz, strain = 100%), the value of G′ decreased from 10.0 kPa to approximately 8.7 kPa, and tanδ increased from 0.02 to 0.24. It can be seen from the rheological experiment that the mechanical stability of the hydrogel was somewhat lost, but the hydrogel system still existed, which was attributed to the stability of the hydrogel network structure. When the amplitude of the oscillating force was reduced (frequency = 1.0 Hz, strain = 10%), the G′ and G″ of SPH could immediately return to their original values, which means that the internal network and mechanical properties of SPH were quickly restored.

### 2.4. Sensing Properties of the Hydrogel

**Figure 5:**
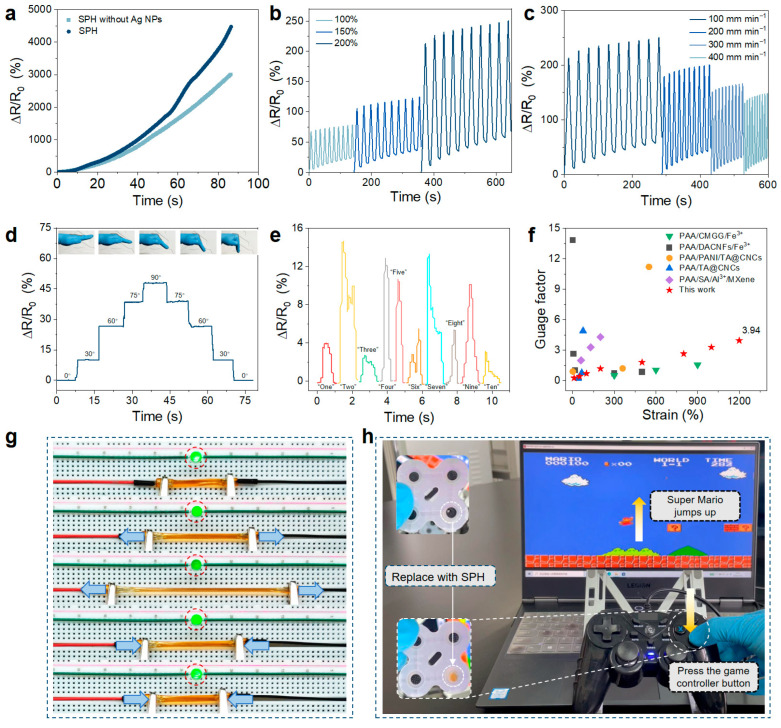
Sensing properties of the hydrogel. (**a**) Strain sensitivity of the hydrogel. (**b**) Curves of the relative resistance change under different strains. (**c**) Curves of the relative resistance change at different stretching speeds. (**d**) Monitoring the multi-angle movement of the finger. (**e**) Variation in the relative resistance over time when the human Adam’s apple is speaking. (**f**) Comparison of the gauge factor between the sensor in this study and previously reported sensors [19,26,27,28,29]. (**g**) The brightness of the LED light changes with the deformation of the hydrogel. (**h**) The hydrogel replaces the conductive adhesive in the buttons of the game controller and successfully controls the computer game.

Due to the presence of Fe^3+^ and lignin–Ag NPs, as well as the tensile strength and compressibility of PAA/OFG, it is speculated that the SPH can be used as a strain sensor. There is a correlation between the resistance change in the SPH and the mechanical strain, so a quantitative study was carried out on this sensor. As shown in Figure 5a, the addition of lignin–Ag NPs can improve the strain sensitivity of the hydrogel, and when the strain is 1200%, the gauge factor is 3.94. Ag NPs can construct nanoscale electronic conduction pathways, and lignin assists in electron transport through conjugate structures and interfacial interactions [22,25]. The Fe^3+^ dynamic network collaboratively regulates ion–electron mixed conductivity, breaking through the limitation of traditional hydrogels that only rely on ionic conduction, and providing a theoretical basis for the design of highly conductive and multifunctional intelligent soft materials. As shown in Figure 5g, the relative resistance change in the SPH can be intuitively observed by evaluating the change in the brightness of the LED light. An LED lamp, hydrogel, and external power supply are connected in series on a breadboard to form a complete conductive path. When the hydrogel deforms, dynamic adjustments in the conductive network structure, charge transport pathways, and interfacial interactions make the resistance positively correlated with the strain, thereby causing the brightness of the LED lamp to change in real time with the different stretching lengths of the hydrogel. As the strain increases, the resistance of the hydrogel increases, and the LED light gradually dims. Generally speaking, compared with traditional rigid strain sensors, stretchable strain sensors have the advantages of conformability and stretchability, solving the problem of mechanical mismatch between electronic devices and the human body. Although the sensitivity of SPH is lower than that of metal-based strain sensors, they still exhibit a large strain sensing range. Ten cycles of tensile loading–unloading measurements were carried out under different strains and speeds to evaluate the robustness of the sensing performance of SPH. The resistance response was stable in all cycles (Figure 5b,c), and SPH showed repeatability and durability.

Since the prepared SPH have high tensile strength and self-healing ability, they were directly used as strain sensors to identify joint movements and vocalizations of organs, so as to construct wearable strain sensors. The SPH sensors were connected to wires, and an electrochemical workstation was used to record the corresponding signals. Figure 5d,e show that the PAA-based sensors are used to detect finger movements and Adam’s apple movements. When the finger connected to the hydrogel sensor bends from the straight state to 0°, 30°, 60°, 75° and 90°, the relative resistance change rate presents a stepped curve (Figure 5d). This indicates that the PAA-based sensor can also be used for data collection and storage of finger movements. At the same time, when the volunteer successively pronounced different Arabic numerals, including “one” to “nine” and “zero”, the hydrogel sensor was able to detect the vibration of the vocal cords (Figure 5e). These results show that the PAA-based hydrogel sensor has excellent micro-strain sensing potential and can be used for precise observation of human movements with a rapid response. To further explore and expand the application field of the hydrogel, the game controller was modified, and the hydrogel was used to replace the conductive adhesive in the controller buttons. After the modification, the game controller can still work normally without delay, further confirming the feasibility of the hydrogel as a conductive material (Figure 5h and Video S1). In addition, the preparation of the multifunctional self-healing hydrogel sensor was compared with the previously reported hydrogel sensors [19,26,27,28,29] in terms of the GF (Figure 5f), which indicates that the PAA hydrogel has superior performance and has great potential in human activity monitoring and personal health diagnosis.

## 3. Conclusions

In summary, a PAA-based hydrogel with superior stretchability and self-healing properties was synthesized by constructing a polymer network incorporating both covalent and multiple non-covalent interactions. The hydrogel demonstrates remarkable mechanical properties, including a stretchability of up to 2000% and a fracture strength of 130 kPa, facilitated by dynamic and reversible metal–ligand coordination interactions. In addition, the reversibility (dissociation and reconstruction) of various non-covalent bonds (H-bonding and metal coordination interaction) endows the hydrogel with excellent self-healing properties (approximately 100%). Furthermore, the incorporation of Fe^3+^ and Ag NPs imparts ionic conductivity, enabling the hydrogel to sense various human movements, including finger gestures and subtle vibrations from the Adam’s apple, and to function as a conductive adhesive in gaming controllers for computer interface control. Therefore, PAA hydrogels exhibit application potential in multiple fields due to their excellent mechanical strength, self-healing ability, and micro-strain sensitivity: in biomedicine, they can serve as advanced wound dressings to monitor healing in real time; in soft robotics, they are suitable for manufacturing high-precision actuators to reduce maintenance requirements; in environmental monitoring, they can be made into highly sensitive sensors adaptable to harsh conditions; and in wearable electronics, they enable the creation of comfortable skin-friendly devices for accurate physiological signal tracking. In the future, further optimization of their properties and functional integration can expand their application prospects.

## 4. Materials and Methods

### 4.1. Materials

AA, FeCl_3_·6H_2_O, APS, and MBA were all purchased from Aladdin Reagent (Shanghai) Co., Ltd. (Shanghai China), with analytical-grade purity. Modified fenugreek galactomannan (OFG) and lignin–Ag NPs (Ag NPs) were prepared according to the methods described in our previously published articles [21,22]. Deionized water was used as the solvent in this study.

### 4.2. Preparation of Hydrogels

The synthesis of PAA/OFG–Fe^3+^ hydrogels involved in situ radical polymerization of AA monomer within an aqueous OFG suspension, using MBA and Fe^3+^ as cross-linkers, and APS as the initiator. Initially, OFG was dissolved in deionized water under room-temperature stirring to form a homogeneous solution with a 0.3% mass fraction. Subsequently, 3.25 g of AA, 0.11 g of FeCl_3_, and 8 mg of APS were added and mixed until uniform. Following this, 2 mg of MBA cross-linker and 200 μL of Ag NPs solution (25 mg/mL) were introduced, and the mixture was rapidly stirred. Oxygen was purged with nitrogen for 1 min before the solution was injected into a custom-made mold composed of two glass plates separated by 2 mm rubber gaskets. The molds were then subjected to polymerization at 50 °C for 2 h to yield the composite hydrogels.

To optimize the influence of various factors on the mechanical properties of hydrogels, different compositions of hydrogels were prepared. In the experiment, the prepared composite hydrogels were named PAA/OFGa (“a” representing the mass percentage of OFG), PAA/OFG-Fe^3+^b (“b” represents the mass content of FeCl_3_), SPH-APSc (“c” represents the mass content of APS), and SPH-MBAd (“d” represents the mass content of the MBA). The total volume of the solution mixture was fixed at 30 mL with deionized water. Tables S1–S4 (Supplementary information) list the detailed components.

### 4.3. Characterization

A Fourier Transform Infrared Spectrometer (FT-IR, Model: Bruker Optics Ltd., Ettlingen, Germany) was employed for qualitative analysis of chemical structure changes in PAA-Fe^3+^ and SPH, with the spectral range set at 400–4000 cm^−1^. X-ray diffraction (XRD) patterns of PAA-Fe^3+^ and SPH were obtained using a Bruker D8 ADVANCE diffractometer (Ettlingen, Germany) under operating conditions of 80 mA and 60 kV, with the diffraction angle (2θ) scanned from 5° to 60°. Young’s modulus data were acquired via a Bruker Icon atomic force microscope (AFM, Bruker Corp., Ettlingen, Germany). The surface morphology of PAA-Fe^3+^ and SPH particles was characterized by a scanning electron microscope (SEM, Model: S-3400 N II, Hitachi, Japan). Prior to testing, samples were dispersed on a metal sample holder using conductive silver paste and subjected to gold sputtering.

The rheological behaviors of PAA-Fe^3+^ and SPHs were investigated using a rotational rheometer (Model: Ares G2, New Castle, DE, USA) with a parallel plate geometry (diameter: 25 mm). The storage modulus (G′) and loss modulus (G″) were measured as functions of angular frequency (ω) within 0.1–100 rad/s. Additionally, the recovery properties of the hydrogels under applied shear forces were tested at ω = 1.0 Hz following the procedure: 10% strain (200 s) → 100% strain (200 s) → 10% strain (200 s) → 100% strain (200 s) → 10% strain (200 s).

### 4.4. Mechanical Testing

Tensile tests were performed using a HESON HS-3004B-S (Shanghai, China) machine equipped with a 100-N load cell. Hydrogel samples with dimensions of 12 mm × 3 mm × 2 mm were tested at a tensile speed of 100 mm/min. The energy dissipation (ΔE) of the self-recovery hydrogel was calculated from the area between loading–unloading curves of cyclic tensile loads, as defined by

ΔE = ∫loading S(μ)dμ − ∫unloading S(μ)d(1)

where S(μ) and μ denote tensile stress and strain, respectively.

The experimental data were independently repeated at least 3 times (*n* ≥ 3), and the sample size (*n*) and standard deviation (SD) of each group of experiments were marked in the Section 2.

### 4.5. Self-Healing Testing

The self-repairing properties of hydrogels were examined by cutting the origin hydrogel and dyeing the hydrogel (blue) into small fragments. With the mold, the size of the hydrogel block was rapidly reconstructed, and the self-healing process of the hydrogel was employed at room temperature without any external force or stimulation. The microscopic morphology of the original hydrogel and the healing hydrogel was observed with a camera.

### 4.6. Fabrication Strain Sensors

An electrochemical workstation (CHI 800 D, Shanghai, China) was used to record relative resistance changes in hydrogel sensors at a constant voltage of 2 V during finger and throat movements. For cyclic strain sensing measurements, the electrochemical workstation was coupled with a tensile testing machine to synchronously record resistance changes during loading. The feasibility of using the hydrogel as a sensor was verified by the brightness variation in an LED lamp and side reactions of a game handle: the LED lamp, hydrogel, and external power supply were connected in series on a breadboard to form a conductive path, and LED brightness was observed by controlling hydrogel deformation. The relative resistance change is defined by Equation (2), and the gauge factor (GF) of the hydrogel is calculated by Equation (3):

ΔR/R_0_ (%) = (R − R_0_)/R_0_ × 100% (2)

GF = (ΔR/R_0_)/ε with ε = (l_T_ − l_0_)/l_0_ (3)

where R_0_ and R are the initial resistance at 0% strain and the real-time resistance at specific strain, respectively; l_0_ and l_T_ are the initial length and the length at time T of the hydrogel.

## Data Availability

The original contributions presented in this study are included in the article. Further inquiries can be directed to the corresponding author.

## Supplementary Materials

The following supporting information can be downloaded at: https://www.mdpi.com/article/10.3390/gels11070475/s1, Figure S1: XRD curves of PAA-Fe^3+^ and SPH. Figure S2: SEM images of PAA-Fe^3+^ and SPH. [Scale bar: 500 μm]. Figure S3: Tensile properties of the hydrogel. (a and b) Influence of the addition of Fe^3+^ on the mechanical properties of the hydrogel. (c) Influence of the addition of APS on the mechanical properties of the hydrogel. (d) Influence of the addition of MBA on the mechanical properties of the hydrogel; Table S1: Schemes of optimized experiment (OFG). Table S2: Schemes of optimized experiment (Fe^3+^). Table S3: Schemes of optimized experiment (APS). Table S4: Schemes of optimized experiment (MBA).; Video S1: Playing games with a controller.

## Abbreviations

The following abbreviations are used in this manuscript:

OFG	Modified fenugreek galactomannan
PAA	Polyacrylic acid
Ag-NPs	Lignin–silver nanoparticles
SPH	Self-healing PAA/OFG-Fe^3+^ hydrogel

## References

[1] Chen K.Y., Xu Y.T., Zhao Y., Li J.K., Wang X.P., Qu L.T. (2023). Recent progress in graphene-based wearable piezoresistive sensors: From 1D to 3D device geometries. Nano Mater. Sci..

[2] Liang Y., Zhao F., Cheng Z.H., Deng Y., Xiao Y., Cheng H., Zhang P., Huang Y., Shao H., Qu L. (2018). Electric power generation via asymmetric moisturizing of graphene oxide for flexible, printable and portable electronics. Energy Environ. Sci..

[3] Wu M., Qiao C.Y., Sui P.F., Luo J., Li Z., Cao Y., Pei R., Peng X., Zeng H. (2025). Stratum corneum-inspired zwitterionic hydrogels with intrinsic water retention and anti-freezing properties for intelligent flexible sensors. Adv. Funct. Mater..

[4] Liu T., Zhang M.Y., Li Z.H., Dou H., Zhang W., Yang J., Wu P., Li D., Mu X. (2025). Machine learning-assisted wearable sensing systems for speech recognition and interaction. Nat. Commun..

[5] Tchantchane R., Zhou H., Zhang S., Alici G. (2025). Fabrication and characterization of a soft and stretchable capacitive strain sensor for hand gesture recognition. IEEE Sens. J..

[6] Zhou X.L., Chen X.L., Yang B., Luo S., Guo M., An N., Tian H., Li X., Shao J. (2025). Advancements in functionalizable metal-organic frameworks for flexible sensing electronics. Adv. Funct. Mater..

[7] Kovacevic B., Jones M., Ionescu C., Walker D., Wagle S., Chester J., Foster T., Brown D., Mikov M., Mooranian A. (2022). The emerging role of bile acids as critical components in nanotechnology and bioengineering: Pharmacology, formulation optimizers and hydrogel-biomaterial applications. Biomaterials.

[8] Yang D., Ni Y., Kong X., Li S., Chen X., Zhang L., Wang Z.L. (2021). Self-healing and elastic triboelectric nanogenerators for muscle motion monitoring and photothermal treatment. ACS Nano.

[9] Li H., Liang Y., Gao G., Wei S., Jian Y., Le X., Lu W., Liu Q., Zhang J., Chen T. (2021). Asymmetric bilayer CNTs-elastomer/hydrogel composite as soft actuators with sensing performance. Chem. Eng. J..

[10] Li Z., Liu P., Ji X., Gong J., Hu Y., Wu W., Wang X., Peng H., Kwok R.T.K., Lam J.W.Y. (2020). Bioinspired simultaneous changes in fluorescence color, brightness, and shape of hydrogels enabled by AIEgens. Adv. Mater..

[11] Tan Y., Yan M.X., Dong H.Q., Wang C., Shao L., Xiao X., Li W., Ji Z., Ling Z. (2025). Biomimetic construction of adhesive, self-healing and antifatigue hydrogels via galactomannan-induced double-network cross-linking for flexible strain sensors. ACS Appl. Polym. Mater..

[12] Tian Y.S., Wei Y., Wang M., Wang J., Li X., Qin X., Zhang L. (2025). Ultra-stretchable, tough, and self-healing polyurethane with tunable microphase separation for flexible wearable electronics. Nano Energy.

[13] Robby A.I., Lee G., Park S.Y. (2019). NIR-induced pH-reversible self-healing monitoring with smartphone by wireless hydrogel sensor. Sens. Actuators B-Chem..

[14] Rao V.K., Shauloff N., Sui X., Wagner H.D., Jelinek R. (2020). Polydiacetylene hydrogel self-healing capacitive strain sensor. J. Mater. Chem. C.

[15] Zhang J., Chen L., Shen B., Mo J., Tang F., Feng J. (2020). Highly stretchable and self-healing double network hydrogel based on polysaccharide and polyzwitterion for wearable electric skin. Polymer.

[16] Ren Z., Ke T., Ling Q., Zhao L., Gu H. (2021). Rapid self-healing and self-adhesive chitosan-based hydrogels by host-guest interaction and dynamic covalent bond as flexible sensor. Carbohydr. Polym..

[17] Wu L., Li L., Pan L., Wang H., Bin Y. (2021). MWCNTsreinforced conductive, self-healing polyvinyl alcohol/carboxymethyl chitosan/oxidized sodium alginate hydrogel as the strain sensor. J. Appl. Polym. Sci..

[18] Wang J., Tang F., Wang Y., Lu Q., Liu S., Li L. (2020). Self-healing and highly stretchable gelatin hydrogel for self-powered strain sensor. ACS Appl. Mater. Interfaces.

[19] Chen W., Bu Y.H., Li D.L., Liu Y., Chen G., Wan X., Li N. (2020). Development of high-strength, tough, and self-healing carboxymethyl guar gum-based hydrogels for human motion detection. J. Mater. Chem. C.

[20] Chen W., Bu Y.H., Li D.L., Liu C., Chen G., Wan X., Li N. (2019). High-strength, tough, and self-healing hydrogel based on carboxymethyl cellulose. Cellulose.

[21] Liu C.J., Ning R.X., Lei F.H., Li P., Wang K., Jiang J. (2022). Study on the structure and physicochemical properties of fenugreek galactomannan modified via octenyl succinic anhydride. Int. J. Biol. Macromol..

[22] Liu C.J., Cheng X.C., Zhang F.L., Lei F., Li P., Wang K., Jiang J. (2023). Preparation and application of galactomannan-based green hydrogels initiated by lignin-Ag NPs. Mater. Today Commun..

[23] Hu F.H., Yu D.H., Gong X., Li Z., Zhao R., Wang Q., Zhang F., Li G., Wang H., Liu W. (2025). Amylopectin-enhanced MXene/gallium hydrogels: High-performance flexible conductive materials for multifunctional sensing, EMI shielding, and energy storage applications. Chem. Eng. J..

[24] Liu X., Chen L.Z., Sufu A., Liu F.F. (2025). Stretchable and self-healing carboxymethyl cellulose/polyacrylic acid conductive hydrogels for monitoring human motions and electrophysiological signals. Int. J. Biol. Macromol..

[25] Gan D., Xing W., Jiang L., Fang J., Zhao C., Ren F., Fang L., Wang K., Lu X. (2019). Plant-inspired adhesive and tough hydrogel based on Ag-Lignin nanoparticles-triggered dynamic redox catechol chemistry. Nat. Commun..

[26] Wang Y., Zhang H., Zhang H., Chen J., Li B., Fu S. (2021). Synergy coordination of cellulose-based dialdehyde and carboxyl with Fe^3+^ recoverable conductive self-healing hydrogel for sensor. Mater. Sci. Eng. C-Mater. Biol. Appl..

[27] Shao C.Y., Meng L., Cui C., Yang J. (2019). An integrated self-healable and robust conductive hydrogel for dynamically self-adhesive and highly conformable electronic skin. J. Mater. Chem. C.

[28] Shao C., Wang M., Meng L., Chang H., Wang B., Xu F., Yang J., Wan P. (2018). Mussel-inspired cellulose nanocomposite tough hydrogels with synergistic self-healing, adhesive, and strain-sensitive properties. Chem. Mater..

[29] Fan C.H., Wang D., Huang J.Y., Ke H.Z., Wei Q.F. (2021). A highly sensitive epidermal sensor based on triple-bonded hydrogels for strain/pressure sensing. Compos. Commun..

