# Supplementary material for: Self-Healing and Tough Polyacrylic Acid-Based Hydrogels for Micro-Strain Sensors
Source: Gels. 2025 Jun 20;11(7):475. doi: 10.3390/gels11070475 (PMC12295542; doi:10.3390/gels11070475)
Supplement: Supplementary file 1 [file gels-11-00475-s001.zip › Supporting Information.pdf]

Supporting Information

Self-Healing and Tough Polyacrylic Acid-Based Hydrogels for Micro-Strain Sensors

Chuanjie Liu[1], Zhihong Liu[2*], and Bing Lu[1*]

[1] School of Chemistry and Chemical Engineering, Beijing Institute of Technology, Beijing 100081, China
[2] Shenzhen Polytechnic University, Guangdong 518055, China
[*] Corresponding author (email: liuzhihong@szpu.edu.cn; blu@bit.edu.cn)

Table S1. Schemes of optimized experiment (OFG).

Sample code	OFG (g)	AA (g)	$FeCl_3·6H_2O$ (g)	APS (mg)	MBA (mg)	H_2O (mL)
PAA/OFG_0	0	6.5	0.11	8	2	30
$PAA/OFG_{0.1}$	0.03	6.5	0.11	8	2	30
$PAA/OFG_{0.3}$	0.09	6.5	0.11	8	2	30
$PAA/OFG_{0.5}$	0.15	6.5	0.11	8	2	30
$PAA/OFG_{1.0}$	0.30	6.5	0.11	8	2	30
$PAA/OFG_{1.5}$	0.45	6.5	0.11	8	2	30

Table S2. Schemes of optimized experiment (Fe^{3+}).

Sample code	OFG (g)	AA (g)	$FeCl_3·6H_2O$ (g)	APS (mg)	MBA (mg)	H_2O (mL)
$PAA/OFG\text{-}Fe^{3+}{}_0$	0.09	6.5	0	8	2	30
$PAA/OFG\text{-}Fe^{3+}{}_{0.05}$	0.09	6.5	0.05	8	2	30
$PAA/OFG\text{-}Fe^{3+}{}_{0.10}$	0.09	6.5	0.10	8	2	30
$PAA/OFG\text{-}Fe^{3+}{}_{0.15}$	0.09	6.5	0.15	8	2	30
$PAA/OFG\text{-}Fe^{3+}{}_{0.20}$	0.09	6.5	0.20	8	2	30
$PAA/OFG\text{-}Fe^{3+}{}_{0.25}$	0.09	6.5	0.25	8	2	30

Table S3. Schemes of optimized experiment (APS).

Sample code	OFG (g)	AA (g)	$FeCl_3·6H_2O$ (g)	APS (mg)	MBA (mg)	H_2O (mL)
$PAA/OFG\text{-}APS_0$	0.09	6.5	0.10	0	2	30
$PAA/OFG\text{-}APS_4$	0.09	6.5	0.10	4	2	30
$PAA/OFG\text{-}APS_8$	0.09	6.5	0.10	8	2	30
$PAA/OFG\text{-}APS_{12}$	0.09	6.5	0.10	12	2	30
$PAA/OFG\text{-}APS_{16}$	0.09	6.5	0.10	16	2	30

Table S4. Schemes of optimized experiment (MBA).

Sample code	OFG (g)	AA (g)	$FeCl_3·6H_2O$ (g)	APS (mg)	MBA (mg)	H_2O (mL)
$PAA/OFG\text{-}MBA_0$	0.09	6.5	0.10	8	0	30
$PAA/OFG\text{-}MBA_1$	0.09	6.5	0.10	8	1	30
$PAA/OFG\text{-}MBA_2$	0.09	6.5	0.10	8	2	30
$PAA/OFG\text{-}MBA_3$	0.09	6.5	0.10	8	3	30
$PAA/OFG\text{-}MBA_4$	0.09	6.5	0.10	8	4	30

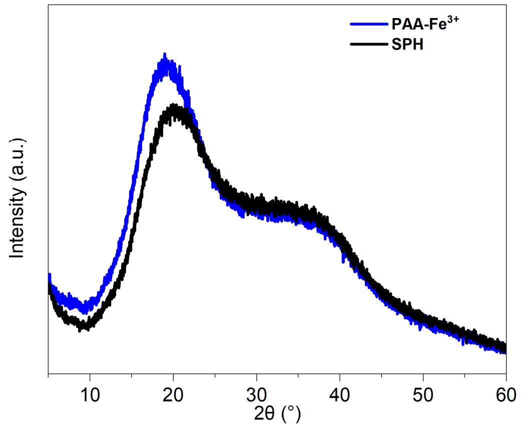

Figure S1. XRD curves of PAA-Fe^{3+} and SPH.

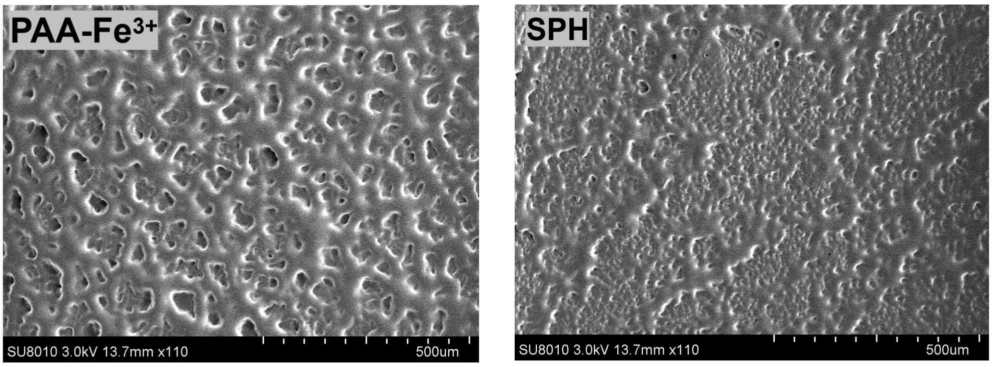

Figure S2. SEM images of PAA-Fe^{3+} and SPH. [Scale bar: 500 μm].

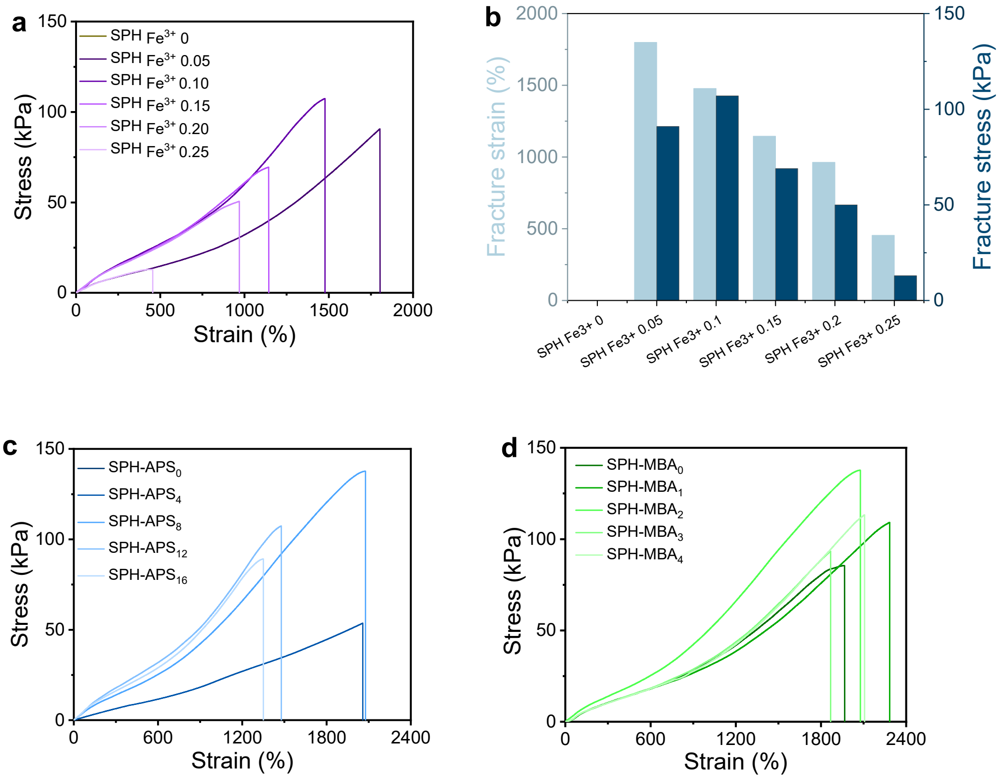

Figure S3. Tensile properties of the hydrogel. (a and b) Influence of the addition amount of Fe^{3+} on the mechanical properties of the hydrogel. (c) Influence of the addition amount of APS on the mechanical properties of the hydrogel. (d) Influence of the addition amount of MBA on the mechanical properties of the hydrogel.
